# Efficacy and Safety of Microvascular Decompression in Patients with Trigeminal Neuralgia Previously Treated with Gamma Knife Stereotactic Radiosurgery

**DOI:** 10.3390/brainsci16090997

**Published:** 2026-09-21

**Authors:** Sebastian Dzierzęcki, Grzegorz Turek, Michał Turek, Zenon Mariak, Wojciech Czyżewski, Jan Gajewski, Jakub Litak, Magdalena Rybaczek, Mateusz Ząbek, Aleksandra Giba, Patrycja Zabrocka, Mirosław Ząbek

**Affiliations:** 1 Department of Radiosurgery, Warsaw Gamma Knife Center, 03-242 Warsaw, Poland; smd@gammaknife.pl; 2 Department of Neurosurgery, Brodno Mazovian Hospital, 03-242 Warsaw, Poland; 3 Department of Neurosurgery, Medical University of Bialystok, 15-089 Bialystok, Poland; zenon.mariak@umb.edu.pl (Z.M.); magdalenarybaczek@interia.pl (M.R.);; 4 Department of Neurosurgery, Maria Sklodowska-Curie National Research Institute of Oncology, 02-781 Warsaw, Poland; wojciech.w.czyzewski@gmail.com; 5 Faculty of Physical Education, Józef Piłsudski University of Physical Education, 01-813 Warsaw, Poland; jan.gajewski@awf.edu.pl; 6 General Surgery Department with Neurosurgery, Poviat Specialist Hospital in Stalowa Wola, 37-450 Stalowa Wola, Poland; 7 Faculty of Medicine, Cardinal Stefan Wyszyński University, 01-938 Warsaw, Poland; 8 Department of General, Vascular, Endocrine, and Transplant Surgery, Medical University of Warsaw, 02-097 Warsaw, Poland; giba.aleksandra99@gmail.com

**Keywords:** trigeminal neuralgia, microvascular decompression, Gamma Knife radiosurgery

## Abstract

**Highlights:**

**What are the main findings?**

- Microvascular decompression provided comparable long-term pain control in patients with and without prior Gamma Knife stereotactic radiosurgery.
- Radiation-related intraoperative tissue changes were present in every previously irradiated patient and made the dissection more demanding, yet were not associated with a higher rate of non-sensory postoperative complications.

**What are the implications of the main findings?**

- Microvascular decompression remains a safe and effective rescue treatment for persistent or recurrent trigeminal neuralgia after unsuccessful Gamma Knife radiosurgery.
- Previous Gamma Knife radiosurgery should not be considered a contraindication to subsequent microvascular decompression in appropriately selected patients.

**Abstract:**

**Background/Objectives**: Gamma Knife stereotactic radiosurgery (GKS) controls classical trigeminal neuralgia (TN) in 70–90% of patients, but pain recurs in a substantial minority, and these patients are then considered for microvascular decompression (MVD). Whether radiation-induced arachnoid thickening, vessel–nerve adhesion and nerve atrophy compromise the safety or efficacy of subsequent MVD remains unresolved, and the available series are small. The objective of this study was to determine whether prior GKS alters the efficacy or the safety of subsequent MVD. **Methods:** We performed a retrospective, two-center cohort study of consecutive patients with classical TN who underwent MVD between 2015 and 2025. Of more than 1571 patients treated with GKS for TN during this period, 35 subsequently underwent MVD for insufficient pain relief (GKS+); they were compared with 60 consecutive patients who underwent primary MVD without prior radiosurgery (GKS−). The primary outcome was pain control on the Barrow Neurological Institute (BNI) scale at last follow-up, dichotomized as good (BNI I–III) or poor (BNI IV–V). Secondary outcomes were postoperative adverse events, medication-free status, intraoperative findings, intradural operative time and length of stay. **Results:** Good long-term pain control was achieved in 31 of 35 GKS+ patients (88.6%) and 55 of 60 GKS− patients (91.7%; *p* = 0.725) over a mean follow-up of 31.2 and 39.7 months, respectively. Radiation-related intraoperative changes—most often arachnoid thickening (62.9%)—were present in all GKS+ patients. Composite adverse events occurred in 31.4% of GKS+ and 36.7% of GKS− patients (*p* = 0.660), but the majority were trigeminal sensory disturbances (22.9% and 25.0%, respectively); non-sensory complications occurred in 3 GKS+ patients (8.6%) and 7 GKS− patients (11.7%; *p* = 0.741). At long-term follow-up, 48.6% of GKS+ and 60.0% of GKS− patients were medication-free (*p* = 0.294). There were no major neurological deficits and no surgery-related deaths in either cohort. **Conclusions:** Prior Gamma Knife radiosurgery did not reduce the efficacy or the safety of subsequent microvascular decompression, despite consistently more demanding intraoperative conditions. MVD remains an appropriate rescue option for persistent or recurrent trigeminal neuralgia after failed radiosurgery.

## 1. Introduction

Trigeminal neuralgia (TN) is characterized by severe, paroxysmal pain affecting one or more divisions of the trigeminal nerve. The prevalence in the general population is estimated to range between 0.03% and 0.3%, with a higher incidence observed among women and individuals over the age of 50 [1,2]. In most cases, TN results from a neurovascular conflict at the root entry zone (REZ) [3,4,5], most commonly due to compression by the superior cerebellar artery (SCA) [6]. First-line treatment consists of pharmacotherapy with anticonvulsants, most commonly carbamazepine or oxcarbazepine [7,8,9,10,11].

When medical treatment fails or becomes intolerable, several interventional options are available, and they differ substantially in invasiveness, durability and infrastructure requirements. Percutaneous destructive procedures directed at the Gasserian ganglion—radiofrequency thermocoagulation, balloon compression and glycerol rhizolysis—are the most widely accessible, since they require only fluoroscopy and can be performed in almost any neurosurgical unit; they provide rapid relief in the great majority of patients but carry a higher rate of sensory loss and a recurrence rate that exceeds 50% at five years [9,10,11,12,13]. Stereotactic radiosurgery (SRS) is the least invasive option but demands dedicated equipment that comparatively few centers possess. Microvascular decompression (MVD) is the only treatment that addresses the presumed cause rather than the symptom, and it offers the most durable relief at the cost of a posterior fossa craniotomy [9,10,14]. The choice among these modalities therefore reflects nerve-conflict anatomy, patient fitness and, in practice, local availability.

Stereotactic radiosurgery, first introduced by Lars Leksell in 1951 [15], enables the precise delivery of a high radiation dose (80–90 Gy) to the trigeminal nerve in the region adjacent to the REZ [16,17,18,19,20]. Although reported efficacy ranges from 70% to 90%, a proportion of patients experience pain recurrence within 6–24 months after the procedure, necessitating surgical intervention [21,22,23].

The evolution of microvascular decompression rests on several key contributions. Walter Dandy first described the relationship between vascular compression of the trigeminal root and trigeminal neuralgia in 1934 [24]. W. James Gardner subsequently developed deliberate decompression of the trigeminal sensory root and emphasized the therapeutic value of relieving neurovascular compression, which he reported with Miklos in 1959, several years before Jannetta′s pioneering microsurgical work [25]. Building on these observations and on his early microsurgical collaboration with Robert W. Rand, Peter Jannetta used the operating microscope to demonstrate arterial compression of the trigeminal nerve at the root entry zone in 1967 and subsequently refined and popularized the modern microvascular decompression technique [26,27,28,29]. The operation is performed through a retrosigmoid approach and consists of separating the offending vessel from the nerve, either with a Teflon^®^ pledget or by vessel transposition [30,31].

In patients previously treated with SRS, MVD may be technically more challenging because of radiation-induced changes, including arachnoid thickening, vessel–nerve adhesions, and alterations of the nerve surface [32,33]. Whether these changes translate into worse pain outcomes or more frequent complications is unclear, because the existing series are limited by small cohorts, short follow-up, or the absence of a contemporaneous comparison group operated by the same surgeons [34,35,36,37,38,39,40].

The aim of this study was to evaluate the efficacy and safety of MVD in patients with TN who failed to achieve adequate pain relief following GKS, to compare these outcomes directly with a contemporaneous cohort undergoing primary MVD at the same centers, and to place both in the context of previously published series.

## 2. Materials and Methods

### 2.1. Study Design and Patient Population

This was a retrospective, observational, two-center cohort study. Patients with TN who underwent MVD between 2015 and 2025 at the neurosurgical departments in Warsaw and Białystok were included. Both centers perform the highest volume of MVD procedures in Poland, and within each department, all procedures were performed by a single surgeon. The two operators (GT and ZM) are linked by a teacher–pupil relationship, which ensures a consistent operative technique across centers and removes surgeon-related variability as a confounder.

The study group consisted of patients previously treated with Gamma Knife SRS (GKS+), while the control group comprised patients undergoing primary MVD (GKS−).

All included patients met the diagnostic criteria for classical TN according to the Headache Classification Committee of the International Headache Society [41], had MRI-confirmed neurovascular conflict [42,43,44], and had failed pharmacological treatment and—in the GKS+ group—radiosurgical treatment. Patients with TN caused by cerebellopontine angle tumors, those lacking complete clinical data, and those with insufficient follow-up were excluded.

Demographic (age, sex), clinical (side, pain distribution, disease duration, type of conflict), intraoperative, and postoperative variables were analyzed, with all data obtained from hospital medical records. Surgical efficacy was assessed using the BNI pain intensity scale [45].

Potential sources of bias included the retrospective design, the absence of blinding in outcome assessment, and variability in documentation detail. Consecutive patient inclusion and a uniform surgical technique were used to minimize selection and information bias. The study size was determined by the number of consecutive eligible patients treated with MVD during the study period; no formal sample size calculation was performed.

### 2.2. Treatment Allocation and Decision-Making

As all GKS+ patients had a neurovascular conflict during surgery, allocation of patients requires additional explanation. A radiologically identifiable conflict is a favorable prognostic factor for MVD as well as for Gamma Knife radiosurgery [46,47]. Therefore, all patients undergoing Gamma Knife radiosurgery presented an anatomical profile associated with a better, not a worse, expected radiosurgical outcome.

Patients treated at the Gamma Knife Centre Warsaw—an institution with more than 16,500 Gamma Knife procedures across all indications, including over 1571 for trigeminal neuralgia during the study period—were allocated according to the center’s internal protocol. After a detailed discussion of the available options, and specifically of the trade-off between the lower immediate morbidity of radiosurgery and the greater durability of decompression, the choice was, in a large proportion of cases, made by the patient rather than by a rigid anatomical threshold.

Referral for MVD followed clinical criteria: no meaningful improvement in pain within six months of GKS, with continued evident distress and pain responding poorly to maximal tolerated pharmacotherapy. Because the Centre routinely offers a second GKS when the therapeutic effect of the initial treatment has waned and neuralgic pain has recurred, referral for open surgery represented a considered escalation rather than a default pathway. This study accordingly asks not who should receive radiosurgery rather than decompression, but whether MVD remains effective and safe once radiosurgery has failed.

### 2.3. Radiosurgical Technique

**Figure 1:**
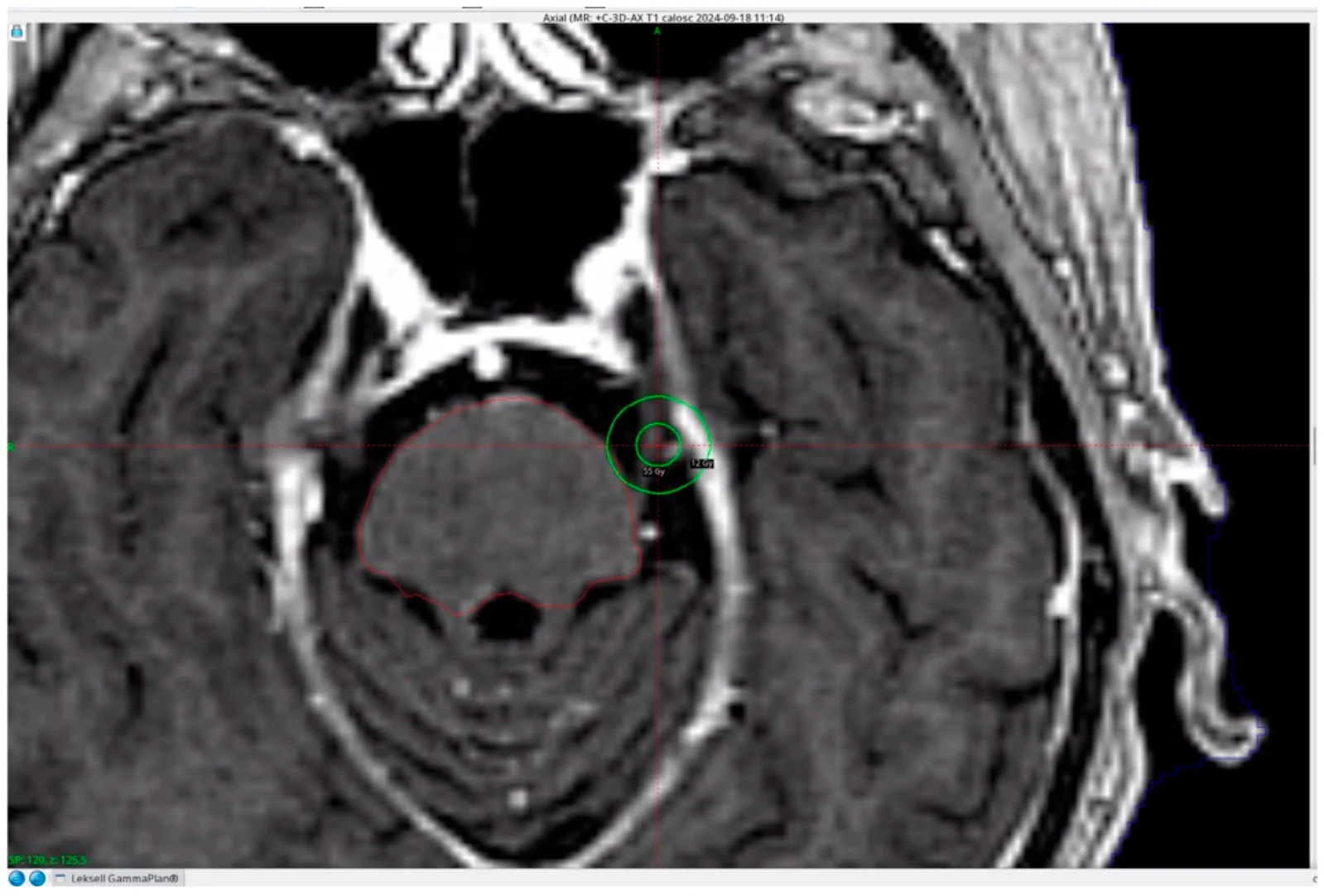
Contrast-enhanced axial T1-weighted magnetic resonance image showing a single 4-mm isocentre applied to the cisternal portion of the left trigeminal nerve. The conflicting vessel is located in the vicinity of the prescribed 85 Gy isodose.

For all patients in the GKS+ cohort, stereotactic radiosurgery was performed using the Leksell Gamma Knife Perfexion (Elekta AB, Stockholm, Sweden). Following stereotactic head frame fixation, patients underwent thin-slice magnetic resonance imaging. The preoperative MRI protocol included three-dimensional T1-weighted images with contrast enhancement and T2 constructive interference in steady-state sequences without contrast. Radiosurgical plans were created using GammaPlan (Elekta). A single 4-mm isocenter was positioned on the cisternal portion of the trigeminal nerve at a distance allowing the 12 Gy isodose to touch the pons (Figure 1). Beam-blocking strategies were applied to minimize radiation exposure to the brainstem, and the volume of brainstem receiving > 16 Gy was kept below 1 mm^3^. Treatment planning was performed in a multidisciplinary fashion with the participation of a neurosurgeon, a radiation oncologist, and a medical physicist.

### 2.4. Surgical Procedure and Intraoperative Monitoring

In each case, the surgery included full exposure of the nerve along its entire course from Meckel’s cave to the pons, followed by meticulous inspection around its entire circumference. This rule was uniformly followed, regardless of whether the conflict with the arterial vessel—which most often involved the SCA—had been previously identified, and decompression was achieved either by arterial transposition or by isolation with a Teflon^®^ pledget. As the superior petrosal vein (SPV) was uniformly spared, its tributaries were followed up to the dorsal aspect of the REZ and, if found in direct contact with the nerve, carefully separated and isolated. This maneuver usually required sharp division of the arachnoid adhesions between the REZ and the SPV, and often revealed other veins in this area, which were coagulated and divided [48].

Intradural operative time (“dura-to-dura time”) was defined as the interval from dural opening to completion of dural closure. Patient positioning, skin incision, soft-tissue dissection, craniotomy and extracranial wound closure were excluded, so that the measure reflects the intradural component of the procedure—arachnoid dissection, identification and mobilization of the offending vessel, separation of vessel–nerve adhesions and completion of the decompression—while minimizing variability related to the extracranial approach and closure.

Intraoperative neurophysiological monitoring (IONM) was not routinely employed during MVD for trigeminal neuralgia in either group during the study period. Cranial nerve protection relied on meticulous microsurgical technique, comprising minimal cerebellar retraction, avoidance of traction on the petrosal venous complex and adjacent cranial nerves, and continuous irrigation throughout the intradural stage. This reflected an indication-specific practice rather than a lack of access to monitoring: facial nerve electromyography with direct electrical stimulation is routinely used at our institution during every microsurgical procedure for vestibular schwannoma and cerebellopontine-angle meningioma, with brainstem auditory evoked potential/auditory brainstem response (BAEP/ABR) monitoring added in patients with serviceable preoperative hearing in whom hearing preservation is an operative objective.

### 2.5. Follow-Up and Outcome Assessment

Postoperative follow-up was performed during scheduled outpatient visits and, when necessary, through structured telephone interviews. Clinical outcomes were evaluated using the BNI pain intensity scale at long-term follow-up. A good outcome was defined as BNI grades I–III, indicating complete pain relief or adequate control with minimal medication; a poor outcome was defined as BNI grades IV–V.

Because BNI grades I and II by definition denote freedom from medication, medication-free status was derived directly from the recorded BNI outcomes and is reported separately from pain control, at both the immediate postoperative assessment and long-term follow-up.

Exact dose modifications among patients who continued antiepileptic treatment were not documented with sufficient consistency in the retrospective source records to permit reliable quantitative analysis; we considered it methodologically inappropriate to reconstruct individual dose reductions retrospectively, and this limitation is stated explicitly below and in Section 4.

Postoperative adverse events were systematically recorded and comprised facial numbness or hypoesthesia, cerebrospinal fluid leakage, postoperative hematoma, wound infection, tinnitus and hearing deterioration. For analysis, events were classified a priori into trigeminal sensory disturbance (facial numbness and hypoesthesia) and non-sensory complications. Sensory disturbance was not excluded from the safety analysis, since new facial numbness and hypoesthesia remain clinically relevant and are commonly reported in published MVD series; it is instead reported as a distinct subgroup, allowing sensory effects attributable to manipulation of the trigeminal nerve to be distinguished from other postoperative morbidity. Both the composite adverse-event rate and the non-sensory complication rate are reported, the former because it permits comparison with published series in which sensory disturbance is included. Postoperative symptoms were actively elicited by direct questioning at each follow-up visit rather than recorded only when spontaneously reported.

### 2.6. Statistical Analysis

Continuous variables were expressed as mean ± standard deviation, and categorical variables as numbers and percentages. The Mann–Whitney U test was used for continuous variables, and Fisher′s exact or chi-square test for categorical variables, to compare the GKS+ and GKS− groups. A *p*-value < 0.05 was considered statistically significant. Analyses were performed using Statistica 13.3 (TIBCO Software Inc., Palo Alto, CA, USA).

## 3. Results

### 3.1. Clinical Characteristics of the Patients

A total of 95 consecutive patients with TN underwent MVD, including 35 previously treated with Gamma Knife radiosurgery (GKS+) and 60 who underwent primary MVD without prior radiosurgery (GKS−).

As shown in Table 1, women predominated in both groups, and patient age was similar, approaching 60 years. Pain more frequently affected the right side of the face. None of the demographic variables differed significantly between groups (*p* > 0.05). Preoperatively, 72 patients (76%) experienced severe pain (BNI IV–V), while 23 (24%) reported moderate pain (BNI I–III), and the distribution of preoperative BNI scores did not differ between cohorts (*p* = 0.405).

Length of hospital stay was shorter in the GKS+ group than in the GKS− group (7.2 ± 2.7 versus 8.8 ± 3.2 days; *p* = 0.009). Although the difference reached statistical significance, its absolute magnitude was 1.6 days and is unlikely to carry clinical meaning; the direction of the difference nevertheless argues against the concern that previously irradiated patients require a more protracted recovery. The reasons why the absolute duration of stay in both cohorts exceeds that reported from North American series are addressed in Section 4.

### 3.2. Surgical Findings

Detailed intraoperative findings are summarized in Table 2. A clear neurovascular conflict was identified in all patients undergoing MVD. The SCA was the most frequent offending vessel, followed by mixed artery–vein and venous compression [5,49]. Arterial conflicts predominated in both groups; however, the relatively large number of categories and the limited sample size within each subgroup precluded a meaningful statistical comparison between the type of vascular conflict and postoperative outcome.

The operative technique consisted of standard microvascular decompression, in which the offending vessel was gently separated from the trigeminal nerve and either insulated with a Teflon^®^ pad or displaced and fastened away from the nerve. In some cases, separation was performed using a thin hemostatic patch, while small arterial or venous branches were coagulated when required to achieve complete decompression and hemostasis.

In the GKS+ group, characteristic radiation-induced changes were observed in every patient. These included arachnoid thickening, dense adhesions between the vessel and the nerve, and nerve atrophy manifested by flattening and thinning of the fibers [32,33]. These radiation-associated changes were accompanied by longer intradural operative times and required more meticulous microsurgical dissection, supporting greater procedural complexity in the GKS+ group: median dura-to-dura time was 37.0 min (IQR 33.0–39.5) versus 29.5 min (IQR 26.0–36.0) after primary MVD (Mann–Whitney U test, *p* < 0.001). Complete decompression was nevertheless achieved in all cases.

### 3.3. Outcomes, Medication Status and Postoperative Adverse Events

Both groups demonstrated comparable outcomes following MVD (Table 3). Good long-term results (BNI I–III) were achieved in 88.6% of the GKS+ cohort and 91.7% of the GKS− cohort, with no significant difference (*p* = 0.725). No patient in the GKS+ cohort presented with severe persistent pain corresponding to BNI V, whereas three such cases occurred in the GKS− group. Mean follow-up was 31.2 months in the GKS+ group and 39.7 months in the GKS− group.

Medication-free status, derived from BNI grades I–II, was achieved immediately after surgery in 28 of 35 GKS+ patients (80.0%) and 43 of 60 GKS− patients (71.7%; *p* = 0.466). At long-term follow-up, the corresponding proportions were 17 of 35 (48.6%) and 36 of 60 (60.0%; *p* = 0.294).

Composite adverse events were recorded in 11 GKS+ patients (31.4%) and 22 GKS− patients (36.7%; *p* = 0.660). Trigeminal sensory disturbance accounted for the majority of these events, occurring in 8 GKS+ patients (22.9%) and 15 GKS− patients (25.0%; *p* = 1.000). Non-sensory complications occurred in 3 GKS+ patients (8.6%) and 7 GKS− patients (11.7%; *p* = 0.741), and comprised postoperative hematoma, tinnitus and hearing deterioration.

Tinnitus was recorded in 5 of 95 patients overall (5.3%), in 2 GKS+ patients (5.7%) and 3 GKS− patients (5.0%; *p* = 1.000). Four of the five episodes were transient and had resolved completely by the first follow-up visit; in one patient, tinnitus persisted and was accompanied by audiometric deterioration from Gardner–Robertson grade I to grade III. Hearing deterioration was otherwise uncommon, recorded in no GKS+ patient and in 2 GKS− patients (3.3%; *p* = 0.533). No major neurological deficits, no cerebrospinal fluid leaks requiring reoperation and no surgery-related deaths occurred in either group; the low event counts nevertheless limit the precision of these comparisons.

## 4. Discussion

The central finding of this study is that prior Gamma Knife radiosurgery did not compromise either the efficacy or the safety of subsequent microvascular decompression, even though radiation-related tissue changes were present in every previously irradiated patient and made the dissection consistently more demanding. Good long-term pain control was achieved in 88.6% of GKS+ patients compared with 91.7% after primary MVD, and non-sensory complications were, if anything, less frequent in the previously irradiated group.

### 4.1. Comparison with Previously Published Series

The comparison with previously published studies was undertaken as a narrative contextual synthesis rather than a systematic review. It did not follow PRISMA methodology and was not designed to generate an independent pooled estimate of treatment effect. The published series are presented to provide clinical context for the findings of the present cohort and should not be interpreted as a systematic review or meta-analysis.

The identified series are summarized in Table 4. Across these reports, the rate of good pain control ranged from 58% to 100% in patients of a mean age near 58 years, among whom women predominated, and the most frequently reported adverse event was facial numbness. Our success rate of 88.6% sits within this range. Chen and Wang and colleagues described a trend toward lower success after MVD in previously irradiated patients, although in neither series did the difference reach significance, and none of the reviewed studies examined the influence of patient sex or age on surgical outcome [34,35,36,37,38,39,40].

Radiosurgical parameters were comparable across the reviewed studies, with doses ranging from 80 to 90 Gy and a mean of approximately 85 Gy. Because of the retrospective design and the absence of standardized reporting, the relationship between radiation dose and MVD outcome could not be analyzed; none of the authors reported a correlation between delivered dose and postoperative results, complication rates, or intraoperative findings.

Intraoperative findings in previously irradiated patients were consistent across series: arachnoid thickening, vessel–nerve adhesion and varying degrees of trigeminal nerve atrophy. Most authors observed no clear relationship between these changes and either complications or pain outcome, although Wang and colleagues reported a significantly higher rate of facial numbness in their GKS+ cohort. In our series, comparable alterations were present in all GKS+ patients and, although they rendered the procedure technically more demanding, they did not translate into a higher complication rate or worse pain control.

Across the published comparative series identified for contextual comparison (Table 5), long-term pain outcomes after MVD were broadly similar in patients with and without previous GKS. Individual studies did not demonstrate a consistent statistically significant disadvantage associated with prior radiosurgery. Given the heterogeneity and non-systematic nature of this narrative comparison, no pooled effect estimate or aggregate between-group statistical test was performed.

### 4.2. Interpretation and Clinical Implications

The question of what constitutes a complication after MVD deserves explicit treatment, because composite rates are otherwise easily misread. A composite adverse-event rate of 31.4% in our GKS+ cohort appears high beside the 9.5% reported by Chen, yet the difference reflects definition and ascertainment rather than surgical performance: our figure includes every recorded trigeminal sensory change, including mild and transient numbness elicited on direct questioning, whereas series reporting single-digit rates generally count only sustained deficits. Sensory disturbance after manipulation of a sensory nerve is an anticipated consequence of the operation rather than an unintended injury, and patients accept it readily when the alternative is uncontrolled neuralgic pain. Judged by non-sensory morbidity, our rates were 8.6% in previously irradiated patients and 11.7% after primary MVD. We would encourage future series to report both figures, since the composite alone is not comparable across studies with differing ascertainment intensity.

Pharmacological burden deserves the same explicit treatment, since the BNI scale captures medication dependence only categorically. Almost half of our GKS+ patients and three-fifths of the GKS− cohort were medication-free at long-term follow-up. These proportions sit within the range reported by comparative prospective studies summarized in the European Academy of Neurology guideline, which describe pain freedom without medication at four to five years in approximately 61–80% of patients after MVD and 33–56% after GKS [50]. Series that assessed drug burden directly report substantial reduction: in a recent MVD cohort of 134 patients, 70.1% reduced antiepileptic medication by at least half and 51.5% discontinued it entirely [51], while after Gamma Knife radiosurgery Rogers and colleagues reported complete discontinuation in 41% with a further 30% halving their intake [52], and a 404-patient multicenter radiosurgical cohort recorded a fall in the mean number of medications from 1.98 to 0.90 [53]. These figures are contextual benchmarks drawn from the literature and are not directly comparable with our own proportions, which were derived from BNI grading rather than from documented dose histories.

Tinnitus is an uncommonly reported outcome after MVD for trigeminal neuralgia, and its occurrence in five of our patients warrants comment. The trigeminal REZ lies in proximity to the cochlear nerve and the internal auditory canal, and cerebellar retraction during a retrosigmoid approach transmits traction to the eighth nerve complex, so transient tinnitus is mechanistically plausible even when hearing is preserved. Four of the five episodes resolved by the first follow-up visit; the single persistent case, accompanied by audiometric deterioration from Gardner–Robertson grade I to grade III, is the one event in this series that would plausibly have been detectable intraoperatively by auditory pathway monitoring. Although routine IONM during MVD for trigeminal neuralgia is not currently mandated by major TN-specific guidelines—in contrast to vestibular schwannoma surgery, for which the Congress of Neurological Surgeons supports monitoring in all cases and the EANO guideline describes it as mandatory [54,55]—a recent systematic review devoted specifically to IONM in TN MVD concluded that monitoring can improve intraoperative safety and provide useful real-time information, while noting that protocols and warning criteria remain insufficiently standardized [56]. That single persistent case, together with this accumulating evidence, has prompted us to move toward routine BAEP/ABR monitoring during MVD in patients with useful preoperative hearing.

Our findings also bear on how rescue treatment should be selected after failed radiosurgery, since MVD is not the only option. Percutaneous rhizotomy—by radiofrequency thermocoagulation, balloon compression or glycerol injection—is more widely available than either radiosurgery or microsurgery, requires neither a craniotomy nor a gamma unit, and is well suited to elderly or medically unfit patients [12,13,57]. Its trade-off is a higher rate of sensory loss, including the risk of anaesthesia dolorosa, and a recurrence rate exceeding 50% within five years. In a patient who has already failed radiosurgery, retains a demonstrable neurovascular conflict on MRI and is fit for general anaesthesia, our data support decompression as the intervention most likely to provide durable relief; in a patient who is unfit, or in whom no conflict is demonstrable, a percutaneous procedure remains the more proportionate choice. We did not perform percutaneous procedures in this cohort, so this comparison rests on published outcomes rather than on our own data.

The mean hospital stay of 7.2 days after MVD in previously irradiated patients and 8.8 days after primary MVD is substantially longer than the two days typically reported from North American centres, and this difference reflects health-system organization rather than surgical morbidity. Within the Polish public system, inpatient episodes are reimbursed as procedure-based packages that create no financial pressure toward early discharge; routine postoperative computed tomography, dressing removal and a documented neurological assessment before discharge are performed as inpatient procedures; and many patients travel considerable distances from regions with limited neurosurgical follow-up, so that discharge is customarily deferred until suture removal or full mobilization. No patient in either cohort remained in hospital because of a complication other than the recorded hematomas. Length of stay in this setting should therefore be read as an artefact of local practice, and international comparisons of this metric are of limited value.

### 4.3. Limitations

This study has several limitations. It was a retrospective analysis without blinded outcome assessment, and differences in follow-up duration between the groups could affect the consistency of BNI evaluation. Intraoperative neurophysiological monitoring was not routinely performed during MVD in the study period, so transient or subclinical electrophysiological changes could not be systematically assessed or compared between the GKS+ and GKS− groups. Exact dose modifications among patients who continued antiepileptic medication were not documented consistently in the source records, so pharmacological burden could be characterized only through the medication-free status derivable from BNI grading. The relationship between radiation dose and the severity of intraoperative changes was not analyzed. The small number of patients in individual subgroups limited any exploration of associations between pain distribution, type of vascular conflict, and surgical result, and the low count of poor outcomes and of non-sensory complications means that the between-group comparisons have limited power to exclude a clinically relevant difference; the absence of a statistically significant difference should not be read as evidence of equivalence. The comparison with previously published series was a narrative contextual synthesis rather than a systematic review, was not PRISMA-compliant, and was not designed to yield a pooled estimate of treatment effect. Finally, all operations were performed by two experienced surgeons in high-volume centers, which may restrict the generalizability of these findings to lower-volume settings.

## 5. Conclusions

This two-center cohort study found no significant difference in pain outcomes or complication rates between microvascular decompression performed after failed Gamma Knife radiosurgery and primary decompression, despite radiation-related tissue changes being present in every previously irradiated patient and rendering the dissection consistently more demanding. Although the sample size limits definitive interpretation, prior radiosurgery should not be regarded as a contraindication to subsequent decompression: in a patient with persistent or recurrent trigeminal neuralgia, a demonstrable neurovascular conflict, and acceptable operative risk, microvascular decompression remains an appropriate and durable rescue option.

## Figures and Tables

**Table 1:** Clinical characteristics of patients.

Variable	GKS+ (*n* = 35)	GKS− (*n* = 60)	*p*
Age (years), mean ± SD	60.8 ± 10.5	58.9 ± 12.0	0.378 *
Sex (male/female)	14 (40.0%)/21 (60.0%)	23 (38.3%)/37 (61.7%)	1.000 #
Side (left/right)	15 (43.0%)/20 (57.0%)	23 (38.3%)/37 (61.7%)	0.826 #
**Pain distribution**			–
V1	0 (0.0%)	0 (0.0%)	
V2	8 (22.9%)	5 (7.7%)	
V3	6 (17.1%)	12 (18.5%)	
V1 + V2	3 (8.6%)	12 (18.5%)	
V2 + V3	11 (31.4%)	20 (30.8%)	
V1 + V2 + V3	7 (20.0%)	11 (18.3%)	
**Preoperative BNI score**			0.405 *
I/II	0 (0.0%)/1 (2.8%)	0 (0.0%)/0 (0.0%)	
III	8 (22.9%)	14 (23.3%)	
IV	16 (45.7%)	23 (38.3%)	
V	10 (28.6%)	23 (38.3%)	
**Disease duration before surgery (months), median (IQR)**	35 (12–48)	28 (12–55)	0.524 *
Length of hospital stay (days), mean ± SD	7.2 ± 2.7	8.8 ± 3.2	0.009 *

GKS+: group with prior Gamma Knife surgery; GKS−: group without Gamma Knife surgery; BNI: Barrow Neurological Institute Pain Intensity Score. # Fisher exact test, two-tailed; * Mann–Whitney U test.

**Table 2:** Surgical findings.

Variable	GKS+ (*n* = 35)	GKS− (*n* = 60)	*p*
**Vascular conflict**			–
SCA	15 (42.9%)	23 (38.3%)	
AICA	2 (5.7%)	1 (1.7%)	
VA/BA	0 (0.0%)	0 (0.0%)	
Vein	10 (28.6%)	17 (28.3%)	
Artery + vein	8 (22.8%)	19 (31.7%)	
**Intradural operative time (“dura-to-dura”), min**			
Median (IQR)	37.0 (33.0–39.5)	29.5 (26.0–36.0)	<0.001 *
Mean ± SD	36.8 ± 4.4	30.4 ± 6.0	
Range (observed)	30–45	20–40	
**Intraoperative findings (GKS+ only)**			
Thickened arachnoid	22 (62.9%)	–	
Adhesion vessel–nerve	6 (17.1%)	–	
Trigeminal nerve atrophy	4 (11.4%)	–	
Atherosclerotic plaque	3 (8.6%)	–	

SCA: superior cerebellar artery; AICA: anterior inferior cerebellar artery; VA: vertebral artery; BA: basilar artery. * Mann–Whitney U test.

**Table 3:** Outcomes, medication status and postoperative adverse events.

Variable	GKS+ (*n* = 35)	GKS− (*n* = 60)	*p*
**Immediate outcome, BNI**			0.840 #
I–III (good outcome)	33 (94.3%)	57 (95.0%)	
IV–V (poor outcome)	2 (5.7%)	3 (5.0%)	
I/II/III	25 (71.4%)/3 (8.6%)/5 (14.3%)	37 (61.7%)/6 (10.0%)/14 (23.3%)	
IV/V	2 (5.7%)/0 (0.0%)	2 (3.3%)/1 (1.7%)	
**Long-term outcome, BNI**			
I–III (good outcome)	31 (88.6%)	55 (91.7%)	0.725 #
IV–V (poor outcome)	4 (11.4%)	5 (8.3%)	0.725 #
I/II/III	11 (31.4%)/6 (17.1%)/14 (40.0%)	33 (55.0%)/3 (5.0%)/19 (31.7%)	
IV/V	4 (11.4%)/0 (0.0%)	2 (3.3%)/3 (5.0%)	
Follow-up (months), mean ± SD	31.2 ± 27.6	39.7 ± 32.1	0.332 *
**Postoperative medication status**			
Immediate medication-free (BNI I–II)	28 (80.0%)	43 (71.7%)	0.466 #
Immediate medication required (BNI III–V)	7 (20.0%)	17 (28.3%)	
Long-term medication-free (BNI I–II)	17 (48.6%)	36 (60.0%)	0.294 #
Long-term medication required (BNI III–V)	18 (51.4%)	24 (40.0%)	
**Adverse events, composite**	11 (31.4%)	22 (36.7%)	0.660 #
**Trigeminal sensory disturbance**	8 (22.9%)	15 (25.0%)	1.000 #
Facial numbness	7 (20.0%)	9 (15.0%)	0.588 #
Facial hypoesthesia	1 (2.9%)	6 (10.0%)	0.250 #
**Non-sensory complications**	3 (8.6%)	7 (11.7%)	0.741 #
Hematoma	1 (2.9%)	2 (3.3%)	1.000 #
Tinnitus	2 (5.7%)	3 (5.0%)	1.000 #
Hearing deterioration	0 (0.0%)	2 (3.3%)	0.533 #

GKS+: group with Gamma Knife surgery; GKS−: group without Gamma Knife surgery; BNI score: The Barrow Neurological Institute Pain Intensity Score; # Fisher exact test 2-tailed; * Mann-Whitney U test; *p* < 0.05.

**Table 4:** Published series of MVD after GKS for trigeminal neuralgia: characteristics and key outcomes.

Study (Year)	Cohort			Clinical Outcome			Adverse Events		Intraoperative Findings	
	*N*	Age (Years)	Sex (M/F)	Good	Bad	Median Follow-Up (Months)	*n* (%)	Details	*n* (%)	Details
Shetter et al. (2005) [34]	6	52	2/4	6	0	25.4	2 (33.3%)	FN 16.7% FNP 16.7%	1 (16.7%)	AP 16.7%
Huang et al. (2006) [36]	8	59.5	3/5	7	1	21	2 (25.0%)	FN 25.0%	1 (12.5%)	AP 12.5%
Sekula et al. (2010) [35]	29	63.5	10/19	25	4	19.8	10 (34.5%)	FN 20.7%	21 (72.4%)	TNA 48.3% AVN 20.7% TA 3.4%
Chen (2012) [37]	42	59.5	22/20	41	1	30	4 (9.5%)	FN 9.5%	34 (81.0%)	TA 35.7% TNA 21.4% AP 14.3% AVN 9.5%
Cheng et al. (2017) [40]	36	51.3 ± 13.1	16/20	33	3	28.5	8 (22.2%)	FN 19.4% CSF leak 2.8%	30 (83.3%)	TNA 36.1% AVN 22.2% TA 16.7% AP 8.3%
Zhao et al. (2018) [38]	32	59.9	13/19	31	1	>60	10 (31.2%)	FN 31.2%	32 (100%)	AP 46.9% TA 31.2% TNA 21.9%
Wang et al. (2022) [39]	19	57.6 ± 9.1	9/10	11	8	36	7 (36.8%)	FN 21.1% CSF leak 5.3% T 5.3% HW 5.3%	14 (73.7%)	TNA 47.4% TA 15.8% AP 15.8% AVN 5.3%
**Present study (2026)**	**35**	**60.8 ± 10.5**	**14/21**	**31**	**4**	**31.2 ± 27.6**	**11 (31.4%)**	**FN 20.0%** **T 5.7%** **FH 2.9%** **H 2.9%**	**35 (100.0%)**	**TA 62.9%** **AVN 17.1%** **TNA 11.4%** **AP 8.6%**

Abbreviations: GKS, Gamma Knife surgery; MVD, microvascular decompression; M/F male/female; CSF, cerebrospinal fluid; FN: facial numbness; FNP: facial nerve palsy; T: tinnitus; HW: hearing worsening; FH: facial hypoesthesia; H: hematoma; AP: atherosclerotic plaque; TNA: trigeminal nerve atrophy; AVN: adhesion vessel–nerve; TA: thickened arachnoid.

**Table 5:** Published comparative series of long-term outcomes after MVD in patients with and without previous GKS.

Author (Year)	Group	*N*	BNI I–III	BNI IV–V	*p* *
Chen (2012) [37]	GKS+	42	41 (98%)	1 (2%)	0.055
	GKS−	67	59 (88%)	8 (12%)	
Cheng et al. (2017) [40]	GKS+	36	33 (92%)	3 (8%)	0.798
	GKS−	58	54 (93%)	4 (7%)	
Wang et al. (2022) [39]	GKS+	19	11 (58%)	8 (42%)	0.135
	GKS−	158	118 (75%)	40 (25%)	
Present study (2026)	GKS+	35	31 (89%)	4 (11%)	0.725
	GKS−	60	55 (92%)	5 (8%)	

Abbreviations: MVD, microvascular decompression; GKS, Gamma Knife stereotactic radiosurgery; GKS+, group with prior Gamma Knife surgery; GKS−, group without prior Gamma Knife surgery; BNI, Barrow Neurological Institute Pain Intensity Score. * Within-study comparison as reported by the original authors. No pooled analysis was performed.

## Data Availability

The data presented in this study are available from the corresponding author upon reasonable request. The data are not publicly available due to privacy and confidentiality restrictions related to patient medical records.

## Abbreviations

The following abbreviations are used in this manuscript:

BNI	Barrow Neurological Institute pain intensity scale
GKS	Gamma Knife stereotactic radiosurgery
GKS+	group with Gamma Knife surgery
GKS−	group without Gamma Knife surgery
MVD	Microvascular decompression
SRS	Stereotactic radiosurgery
TN	Trigeminal neuralgia
FN	facial numbness
FNP	facial nerve palsy
T	tinnitus
HW	hearing worsening
FH	facial hypoesthesia
H	hematoma
AP	atherosclerotic plaque
TNA	trigeminal nerve atrophy
AVN	adhesion vessel-nerve
TA	thickened arachnoid
